# A review of Leuctridae (Insecta, Plecoptera) in Wuyi Mountains, China

**DOI:** 10.3897/BDJ.10.e86735

**Published:** 2022-07-21

**Authors:** Yu-Ben Yang, Bin-Qing Zhu, Abdur Rehman, Yu-Zhou Du

**Affiliations:** 1 School of Horticulture and Plant Protection & Institute of Applied Entomology, Yangzhou University, Yangzhou, 225009, China School of Horticulture and Plant Protection & Institute of Applied Entomology, Yangzhou University Yangzhou, 225009 China; 2 Nanjing institute of Environmental Sciences, Ministry of Ecology and Environment/State Environmental Protection Scientific Observation and Research Station for Ecological Environment of Wuyi Mountains/Biodiversity Comprehensive Observation Station for Wuyi Mountains/State Environmental Protection Key Laboratory on Biosafety, Nanjing 210042, China Nanjing institute of Environmental Sciences, Ministry of Ecology and Environment/State Environmental Protection Scientific Observation and Research Station for Ecological Environment of Wuyi Mountains/Biodiversity Comprehensive Observation Station for Wuyi Mountains/State Environmental Protection Key Laboratory on Biosafety Nanjing 210042 China; 3 Joint International Research Laboratory of Agriculture and Agri-Product Safety, the Ministry of Education, Yangzhou University, Yangzhou 225009, China Joint International Research Laboratory of Agriculture and Agri-Product Safety, the Ministry of Education, Yangzhou University Yangzhou 225009 China

**Keywords:** Plecoptera, Leuctridae, new record, Wuyi Mountains, China

## Abstract

**Background:**

Wuyi Mountains are located in the northern Oriental Region and the edge of the southern Palaearctic Region. They have a unique geographical location, complex landform and superior climatic conditions, providing a good ecological environment for Leuctridae species. However, due to the damage of some holotypes in the 20^th^ century, limited drawings and lack of colour figures, it is necessary to reorganise and supplement the preserved Leuctridae specimens from Wuyi Mountains.

**New information:**

In this study, we found that there are twelve species of Leuctridae recorded in Wuyi Mountains, accounting for about 20% of the recorded species of Leuctridae in China. These records include two genera and five new distribution records species: one species of the genus *Paraleuctra* Hanson, 1941: *Paraleuctraorientalis* (Chu 1928) and eleven species of the genus *Rhopalopsole* Klapálek, 1912, including five new distribution records to Wuyi Mountains: *Rhopalopsolefengyangshanensis* Yang, Shi & Li, 2009; *Rhopalopsolesinensis* Yang & Yang, 1993; *Rhopalopsoleyangdingi* Sivec & Harper, 2008; *Rhopalopsoleflata* Yang & Yang, 1995; *Rhopalopsolebasinigra* Yang & Yang, 1995. Now a total of twelve species of Leuctridae have been recorded from Wuyi Mountains, Fujian Province of south-eastern China. In this paper, we also provide a key to the male, new images and some notes of these twelve species, except *Rhopalopsolerecurvispina* (Wu, 1949) and *Rhopalopsolespiniplatta* (Wu, 1949). We failed to collect these two species and we regard *R.recurvispina* as a nomen dubium, because there are no distinctive features that can be used to distinguish this species.

## Introduction

At present, there are more than 380 species of Leuctridae in 17 genera of two subfamilies recorded in the world and 70 species of Leuctridae in four genera are recorded in China, which are distributed in different provinces and regions (Qian and Du 2011, Qian and Du 2012a, Qian and Du 2012b, Du and Qian 2012, Qian and Du 2013, Qian et al. 2014, Yang et al. 2015, Qian and Du 2017, Chen and Du 2017, Li et al. 2017, Mo et al. 2018, Chen 2019, Yang and Du 2021a, Yang and Du 2021b, Yang et al. 2021, Yang and Du 2022a, Yang and Du 2022b, Yang et al. 2022, Chen et al. 2022, Li et al. 2022, DeWalt et al. 2022).

Wuyi Mountains are located in the northern Palaearctic Region and the edge of the southern Oriental Region. They cover an area of 60 km^2^ and the altitude is between 180 m and 720 m. They belong to the middle subtropical monsoon climate and the zonal vegetation is well preserved. The length of the famous Jiuqu River is 9.5 km, thousands of streams having been formed based on this river, providing a good ecological environment for Leuctridae species (Shen 2015). In order to further reveal the potential Leuctridae biodiversity and better understand the composition of Leuctridae in Wuyi Mountains, we visited Wuyi Mountains for investigations many times, we consulted most of the literature and examined a large number of specimens. We found that there are 12 species of Leuctridae recorded in Wuyi Mountains. In this paper, we also provide a key, new images and some notes of these 12 species, except *Rhopalopsolerecurvispina* (Wu, 1949) and *Rhopalopsolespiniplatta* (Wu, 1949). We failed to collect these two species and we regard *R.recurvispina* as a nomen dubium.

## Materials and methods

Specimens in this study were collected by hand and preserved in 75% ethanol. Morphological details were examined with a Leica MZAPO microscope. Color illustrations were taken with a KEYENCE VHX-5000. All specimens used in this study are deposited in the Insect Collection of Yangzhou University (ICYZU), Jiangsu Province, China. The morphological terminology follows Sivec et al. (2008).

## Taxon treatments

### Paraleuctra orientalis

(Chu, 1928)

448F9773-BABD-518F-8845-7922E8A88AA7

Genus *Paraleuctra*: Hanson (1941): 57.
Leuctra orientalis : Chu (1928): 87.
Rhopalopsole orientalis : Illies (1966): 118.
Paraleuctra orientalis : Zwick (1973): 410.
Synonym: *Paraleuctrasinica*: Yang and Du (2022b): 4.
Synonym: *Paraleuctratianmushana*: Yang and Du (2022b): 5.

#### Materials

- **Type status:** Other material. **Occurrence:** recordedBy: Yang Yu-Ben; individualCount: 1; sex: male; lifeStage: adult; occurrenceStatus: present; **Taxon:** scientificName: *Paraleuctraorientalis* Chu, 1928; kingdom: Animalia; phylum: Arthropoda; class: Insecta; order: Plecoptera; family: Leuctridae; taxonRank: species; taxonomicStatus: accepted; **Location:** country: China; countryCode: CN; stateProvince: Fujian Province; locality: Wuyi Mountains (Matouyan); minimumElevationInMeters: 303; verbatimLatitude: 27°39′33″N,; verbatimLongitude: 117°57′50″E; **Identification:** identifiedBy: Yang Yu-Ben & Du Yu-Zhou; **Event:** year: 2021; month: 3; day: 10; verbatimEventDate: 10-03-2021; **Record Level:** language: En; institutionCode: ICYZU; basisOfRecord: PreservedSpecimen.

#### Description

##### Diagnosis and remarks

**Figure 1.**
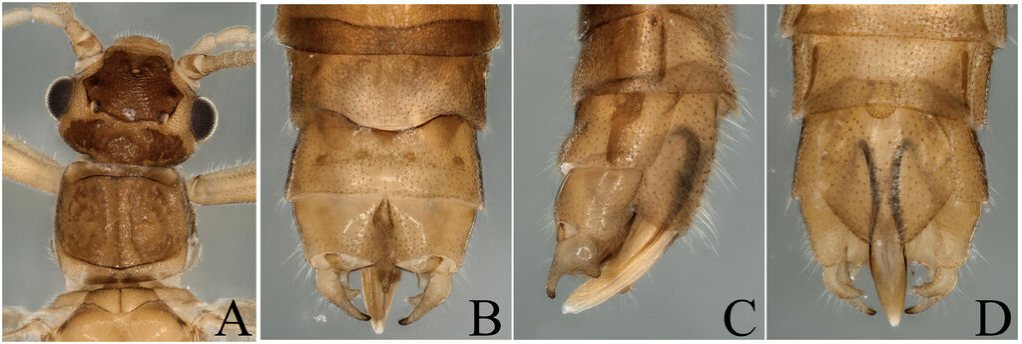
*Paraleuctraorientalis* (Chu, 1928). **A** Male head and pronotum, dorsal view; **B** Male terminalia, dorsal view; **C** Male terminalia, lateral view; **D** Male terminalia, ventral view.

The specimens we collected have the typical terminalia of *P.orientalis*, cerci somewhat sclerotized, strongly forked into two sharp prongs; upper prong longer than lower prong; a spine present near base of upper prong, projecting backwards, so we define them as *P.orientalis* (Fig. 1). The heads of the specimens have strongly sclerotized parts on the ocelli area and the hind margin (Fig. 1A). As the colour and sclerotized patterns bear some variations with the original descriptions, we think the variations are intraspecific.

##### Type locality

China, Zhejiang Province (Lin–an).

#### Distribution

China (Henan, Anhui, Zhejiang, Fujian, Hubei, Hunan, Shaanxi, Sichuan, Yunan, Gansu); Russia (Siberia).

### Rhopalopsole recurvispina

(Wu, 1949), nom. dubium

E8275A28-B972-53C8-8561-3F33497F4848

Genus *Rhopalopsole*: Klapálek (1912): 348.
Leuctra recurvispina : Wu (1949): 252.
Rhopalopsole recurvispina : Illies (1966): 118.

#### Description

##### Material examined

No specimen of this species was found.

##### Diagnosis and remarks

**Figure 2.**
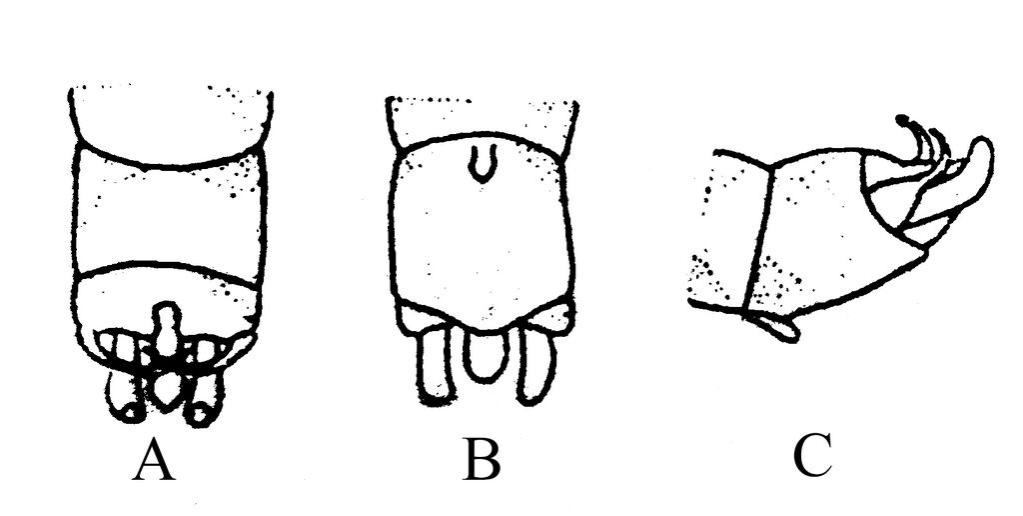
*Rhopalopsolerecurvispina* (Wu, 1949), nom. dubium. **A** Male terminalia, dorsal view; **B** Male terminalia, ventral view; **C** Male terminalia, lateral view. Modified from Wu (1949).

Type of *R.recurvispina* from 1949 is destroyed or lost (Wu 1962). The description in the original literature is not detailed and the drawing is very poor (Fig. 2). The distinguishing characteristics of this species are also not sufficient: there is no obvious sclerotized area on tergum 9 of this species (Fig. 2A). We therefore regard *R.recurvispina* as a nomen dubium after we failed many times to collect this species from the type locality.

##### Type locality

China, Fujian Province (Ta–chu–luan, Shao–wu).

#### Distribution

China (Fujian).

### Rhopalopsole spiniplatta

(Wu, 1949)

9E0A164E-EAAE-56E0-A751-6679911EFE4F

Leuctra spiniplatta : Wu (1949): 252.
Rhopalopsole spiniplatta : Illies (1966): 119.

#### Description

##### Material examined

No specimen of this species was found.

##### Diagnosis and remarks

**Figure 3.**
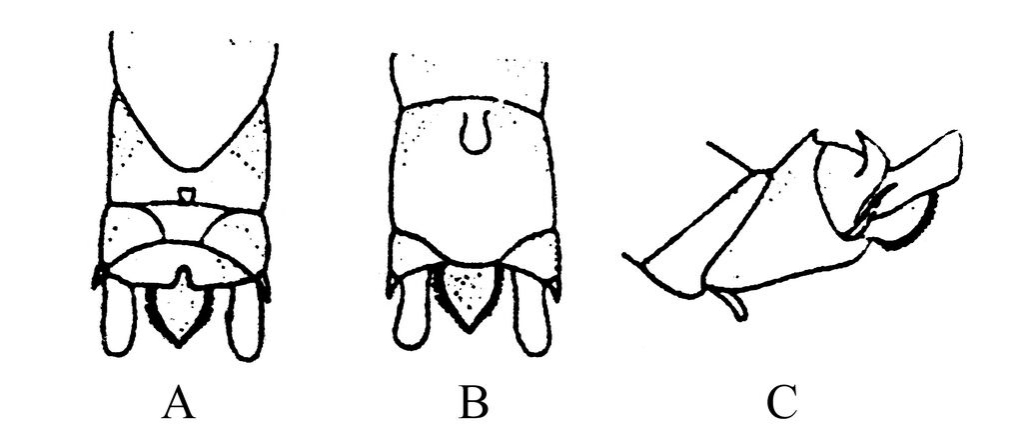
*Rhopalopsolespiniplatta* (Wu, 1949). **A** Male terminalia, dorsal view; **B** Male terminalia, ventral view; **C** Male terminalia, lateral view. Modified from Wu (1949).

Type of *R.spiniplatta* from 1949 is destroyed or lost (Wu 1962). The description in the original literature is not detailed and the drawing is very poor (Fig. 3). However, the distinguishing characteristics of this species are unusual: subanal lobes bordered with black spines and short processes on lateral projections (Fig. 3A and B). We will try to collect the species in the next trip; this could resolve the exact identity of this species.

##### Type locality

China, Fujian Province (Ta–chu–luan, Shao–wu).

#### Distribution

China (Fujian).

### Rhopalopsole fengyangshanensis

Yang, Shi & Li, 2009

FACC4D73-B7F0-5A2B-B815-7B2658E147B9

Rhopalopsole fengyangshanensis : Yang et al. (2009): 193.

#### Materials

- **Type status:** Other material. **Occurrence:** recordedBy: Chen Zhi-Ten, Shen Yue; individualCount: 3; sex: male; occurrenceStatus: present; **Taxon:** scientificName: *Rhopalopsolefengyangshanensis* Yang, Shi & Li, 2009; kingdom: Animalia; phylum: Arthropoda; class: Insecta; order: Plecoptera; family: Leuctridae; taxonRank: species; taxonomicStatus: accepted; **Location:** country: China; countryCode: CN; stateProvince: Fujian Province; locality: Wuyi Mountains (Tongmucun); minimumElevationInMeters: 478; verbatimLatitude: 27°44′51″N; verbatimLongitude: 117°40′39″E; **Identification:** identifiedBy: Yang Yu-Ben; **Event:** year: 2017; month: 5; day: 14; verbatimEventDate: 14-05-2017; **Record Level:** language: En; institutionCode: ICYZU; basisOfRecord: PreservedSpecimen.

#### Description

##### Diagnosis and remarks

**Figure 4.**
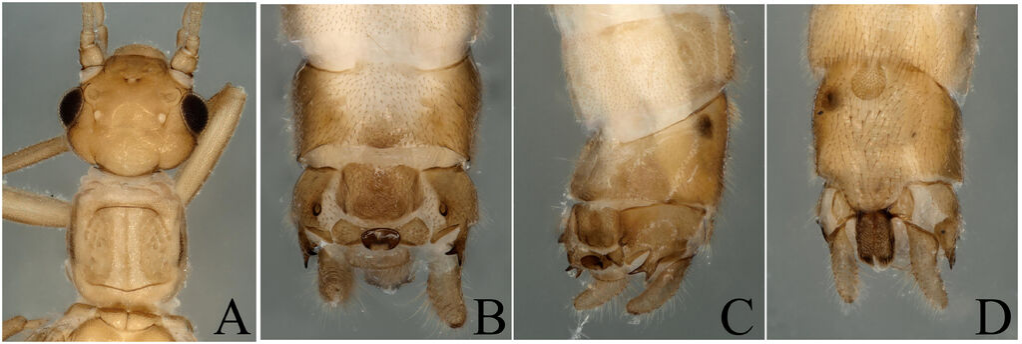
*Rhopalopsolefengyangshanensis* Yang, Shi & Li, 2009. **A** Male head and pronotum, dorsal view; **B** Male terminalia, dorsal view; **C** Male terminalia, lateral view; **D** Male terminalia, ventral view.

The report of this species is a new distribution record to Wuyi Mountains. We think this species belongs to the *R.vietnamica* west group as proposed by Sivec et al. (2008). Head dark brown, slightly wider than pronotum; compound eyes dark; antennae and mouth-parts brown (Fig. 4A). Tergum 9 possesses a small field on its hind margin. Tergum 10 possesses a central plate and two sclerotized bands on each side of the plate. Lateral projections of tergum 10 typically ending in a forked process, inner side has a finger-like ornamentation. Epiproct thick, hook-like, in top view, very blunt (Fig. 4B and C).

##### Type locality

China, Zhejiang Province (Fengyang Mountain, Fengyanghu).

#### Distribution

China (Zhejiang, Fujian, Jiangxi).

### Rhopalopsole intonsa

Qian & Du, 2012

CB6A952F-8495-5CDA-9669-EE1E588BCF0E

Rhopalopsole intonsa : Qian and Du (2012a): 18.

#### Materials

- **Type status:** Other material. **Occurrence:** recordedBy: Xue Hai-Yang (ICYZU); individualCount: 5; sex: Male; occurrenceStatus: present; **Taxon:** scientificName: *Rhopalopsoleintonsa* Qian & Du 2012; kingdom: Animalia; phylum: Arthropoda; class: Insecta; order: Plecoptera; family: Leuctridae; taxonRank: species; taxonomicStatus: accepted; **Location:** continent: Asia; country: China; countryCode: CN; stateProvince: Jiangxi; locality: Wuyi Mountains; **Identification:** identifiedBy: Yang Yu-Ben; **Event:** year: 2008; month: 10; day: 7; verbatimEventDate: 07-10-2008; **Record Level:** language: En; institutionCode: ICYZU; basisOfRecord: PreservedSpecimen.

#### Description

##### Diagnosis and remarks

**Figure 5.**
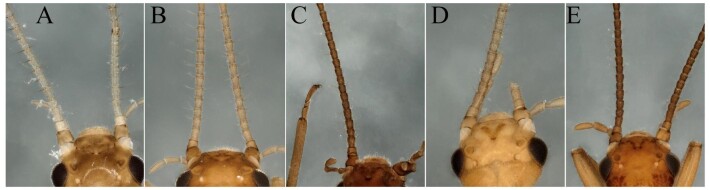
Male antennae, dorsal view. **A** *Rhopalopsoleintonsa* Qian & Du, 2012; **B** *Rhopalopsolesinensis* Yang & Yang, 1993; **C** *Rhopalopsoleampulla* Du & Qian, 2011; **D** *Rhopalopsoleexiguspina* Du & Qian, 2011; **E** *Rhopalopsolememorabilis* Qian & Du, 2012.

**Figure 6.**
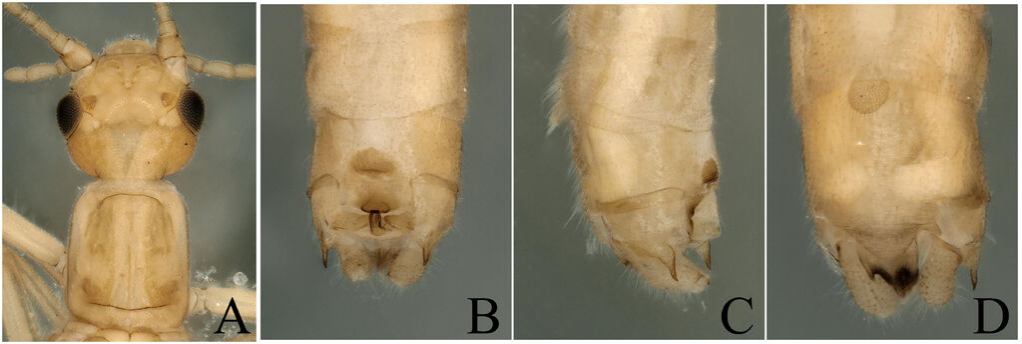
*Rhopalopsoleintonsa* Qian & Du, 2012. **A** Male head and pronotum, dorsal view; **B** Male terminalia, dorsal view; **C** Male terminalia, lateral view; **D** Male terminalia, ventral view.

This species was well described by Qian and Du (2012a). Additionally, we found long hairs on the antennae (Figs 5, 6), which are only found in *R.sinensis* Yang & Yang, 1993, *R.ampulla* Du & Qian, 2011, *R.exiguspina* Du & Qian, 2011 and *R.memorabilis* Qian & Du, 2012 in this genus. In this paper, we show the new images of the species to facilitate terminalia identification (Fig. 6).

##### Type locality

China, Jiangxi Province (Wuyi Mountains).

#### Distribution

China (Jiangxi, Zhejiang).

### Rhopalopsole sinensis

Yang & Yang, 1993

594174A1-50AA-5989-B786-03B3B7671B2C

Rhopalopsole sinensis : Yang and Yang (1993): 236.
Synonym: *Rhopalopsolefurcata*: Yang and Du (2021a): 532.
Synonym: *Rhopalopsolehongpingana*: Yang and Du (2021a): 533.
Synonym: *Rhopalopsoleningxiana*: Yang and Du (2021a): 534.

#### Materials

- **Type status:** Other material. **Occurrence:** recordedBy: Chen Zhi-Ten, Shen Yue (ICYZU); individualCount: 5; sex: Male; occurrenceStatus: present; disposition: ICYZU; **Taxon:** scientificName: *Rhopalopsolesinensis* Yang & Yang, 1993; kingdom: Animalia; phylum: Arthropoda; class: Insecta; order: Plecoptera; family: Leuctridae; taxonRank: species; taxonomicStatus: accepted; **Location:** continent: Asia; country: China; countryCode: CN; stateProvince: Fujian; locality: Wuyi Mountains (Tongmucun); minimumElevationInMeters: 478; verbatimLatitude: 27°44′51″N; verbatimLongitude: 117°40′39″E; **Identification:** identifiedBy: Yang Yu-Ben & DU Yu-Zhou; **Event:** year: 2017; month: 05; day: 14; verbatimEventDate: 14-05-2017; **Record Level:** language: En; institutionCode: ICYZU; basisOfRecord: PreservedSpecimen.

#### Description

##### Diagnosis and remarks

**Figure 7.**
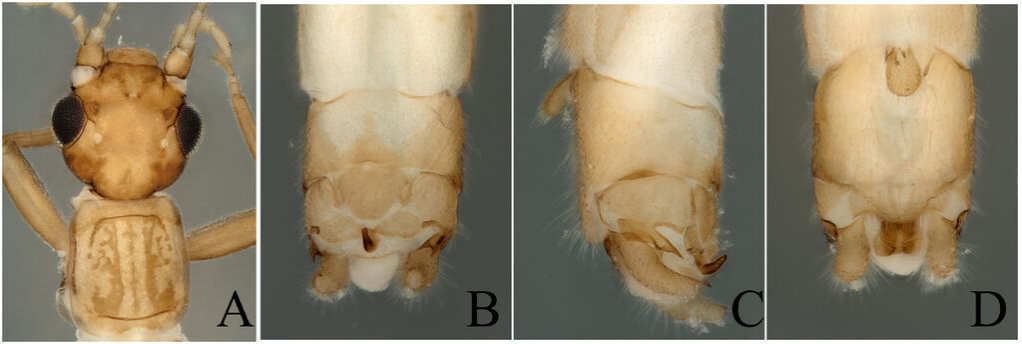
*Rhopalopsolesinensis* Yang & Yang, 1993. **A** Male head and pronotum, dorsal view; **B** Male terminalia, dorsal view; **C** Male terminalia, lateral view; **D** Male terminalia, ventral view.

The report of this is a new distribution record to Wuyi Mountains. *R.sinensis* belongs to *R.vietnamica* group according to Sivec et al. (2008). In China, this species is widespread in southern provinces. The characteristic of long hairs on the antennae can clearly distinguish this species from other similar species in this group (Fig. 5B); other characteristics are shown in Fig. 7.

##### Type locality

China, Guizhou Province (Maolan).

#### Distribution

China (Guangdong, Zhejiang, Fujian, Hubei, Hunan, Henan, Guizhou, Jiangxi, Shaanxi, Sichuan, Guangxi, Ningxia, Yunnan); Vietnam (Laocai).

### Rhopalopsole yangdingi

Sivec & Harper, 2008

D831482C-EA43-5C3B-A27B-3026484B0CB8

Rhopalopsole yangdingi : Sivec et al. (2008): 109.

#### Materials

- **Type status:** Other material. **Occurrence:** recordedBy: Chen Zhi-Ten, Shen Yue (ICYZU); individualCount: 3; sex: male; occurrenceStatus: present; disposition: ICYZU; **Taxon:** scientificName: *Rhopalopsoleyangdingi* Sivec & Harper, 2008; kingdom: Animalia; phylum: Arthropoda; class: Insecta; order: Plecoptera; family: Leuctridae; taxonRank: species; taxonomicStatus: accepted; **Location:** continent: Asia; countryCode: CN; stateProvince: Fujian; locality: Wuyi Mountains (Tongmucun); maximumElevationInMeters: 478; verbatimLatitude: 27°44′51″N,; verbatimLongitude: 117°40′39″E; **Identification:** identifiedBy: Yang Yu-Ben & Du Yu-Zhou; **Event:** year: 2017; month: 05; day: 14; verbatimEventDate: 14-05-2017; **Record Level:** language: En; institutionCode: ICYZU; basisOfRecord: PreservedSpecimen.

#### Description

##### Diagnosis and remarks

**Figure 8.**
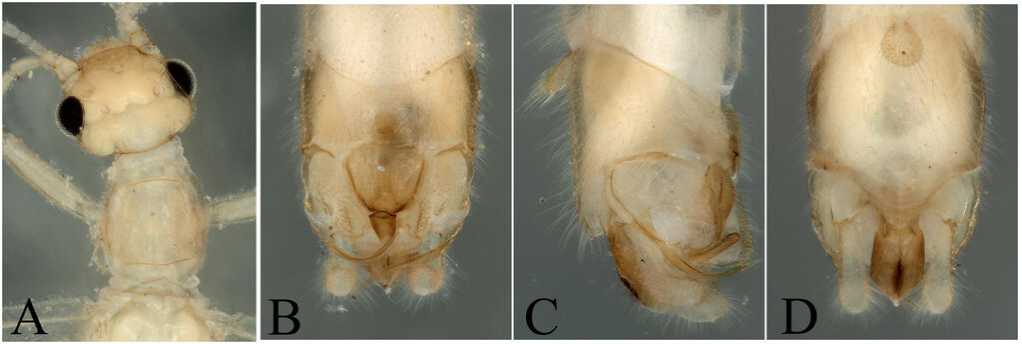
*Rhopalopsoleyangdingi* Sivec & Harper, 2008. **A** Male head and pronotum, dorsal view; **B** Male terminalia, dorsal view; **C** Male terminalia, lateral view; **D** Male terminalia, ventral view.

The report of this species is a new distribution record to Wuyi Mountains and had been well described by Sivec et al. (2008) and we defined our specimens as *R.yangdingi* after we checked the holotype of this species. Head pale brown, slightly wider than pronotum; compound eyes dark; antennae and mouth-parts pale brown (Fig. 8A). Tergum 10 bearing a large central plate and the hind margin strongly sclerotized. Epiproct stocky, terminating in a trilobed tip, the middle is round, two other lobes are corners of epiproct (Fig. 8).

##### Type locality

China, Jiangxi Province (Dayue Mountain).

#### Distribution

China (Jiangxi, Fujian).

### Rhopalopsole flata

Yang & Yang, 1995

918C3D71-4FB3-5DDA-8B52-614C862C8C86

Rhopalopsole flata : Yang and Yang (1995): 61.

#### Materials

- **Type status:** Other material. **Occurrence:** recordedBy: Yang Yu-Ben (ICYZU); individualCount: 3; sex: male; lifeStage: adult; occurrenceStatus: present; **Taxon:** scientificName: *Rhopalopsoleflata* Yang & Yang, 1995; kingdom: Animalia; phylum: Arthropoda; class: Insecta; order: Plecoptera; family: leuctridae; taxonRank: species; taxonomicStatus: accepted; **Location:** continent: Asia; country: China; countryCode: CN; stateProvince: Fujian; locality: Wuyi Mountains (Matouyan); minimumElevationInMeters: 303; verbatimLatitude: 27°39′33″N,; verbatimLongitude: 117°57′50″E; **Identification:** identifiedBy: Yang Yu-ben & Du Yu-Zhou; **Event:** year: 2021; month: 3; day: 10; verbatimEventDate: 10-03-2021; **Record Level:** language: En; institutionCode: ICYZU; basisOfRecord: PreservedSpecimen.

#### Description

##### Diagnosis and remarks

**Figure 9.**
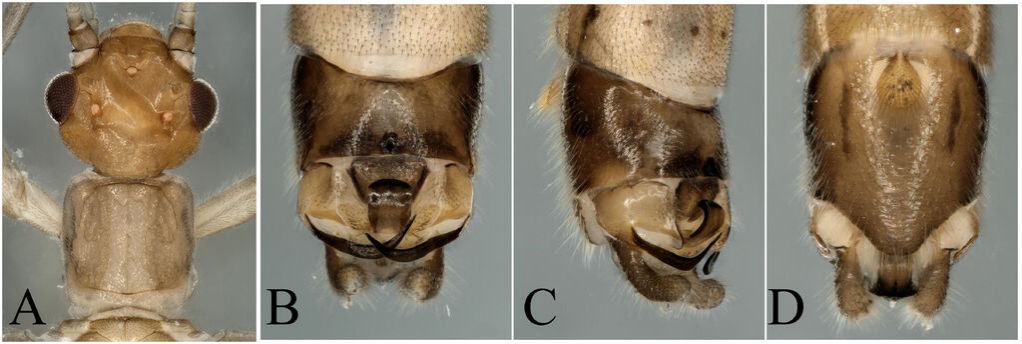
*Rhopalopsoleflata* Yang & Yang, 1995. **A** Male head and pronotum, dorsal view; **B** Male terminalia, dorsal view; **C** Male terminalia, lateral view; **D** Male terminalia, ventral view.

The report of this species is a new distribution record to Wuyi Mountains. Head brown, slightly wider than pronotum; compound eyes dark brown; antennae and mouth-parts brown (Fig. 9A). This species can be easily distinguished by the epiproct almost as wide as the central plate of tergum 10 (Fig. 9B). However, the hind margin of tergum 9 strongly sclerotized (Fig. 9B), this characteristic is not consistent with original descriptions; we think this variation should be the interspecific difference.

##### Type locality

China, Zhejiang Province (Baishanzu Mountain).

#### Distribution

China (Guangdong, Zhejiang, Anhui, Fujian).

### Rhopalopsole wuyishanensis

Yang & Du, 2021

818EC8A1-987C-56D8-BFCC-E2CF07513294

Rhopalopsole wuyishanensis : Yang et al. (2021): 144–145.

#### Materials

- **Type status:** Other material. **Occurrence:** recordedBy: Huo Qing-Bo and Zhu Bin-Qing (ICYZU); individualCount: 6; sex: male; lifeStage: adult; occurrenceStatus: present; **Taxon:** scientificName: *Rhopalopsolewuyishanensis* Yang & Du, 2021; kingdom: Animalia; phylum: Arthropoda; class: Insecta; order: Plecoptera; family: Leuctridae; taxonRank: species; taxonomicStatus: accepted; **Location:** continent: Asia; country: China; stateProvince: Fujian; locality: Wuyi Mountains; minimumElevationInMeters: 726; verbatimLatitude: 27°44′46″N; verbatimLongitude: 27°44′46″N; **Identification:** identifiedBy: Yang Yu-Ben & Du Yu-Zhou; **Event:** year: 2021; month: 4; day: 13; verbatimEventDate: 13-04-2021; **Record Level:** language: En; institutionCode: ICYZU; basisOfRecord: PreservedSpecimen.

#### Description

##### Diagnosis and remarks

**Figure 10.**
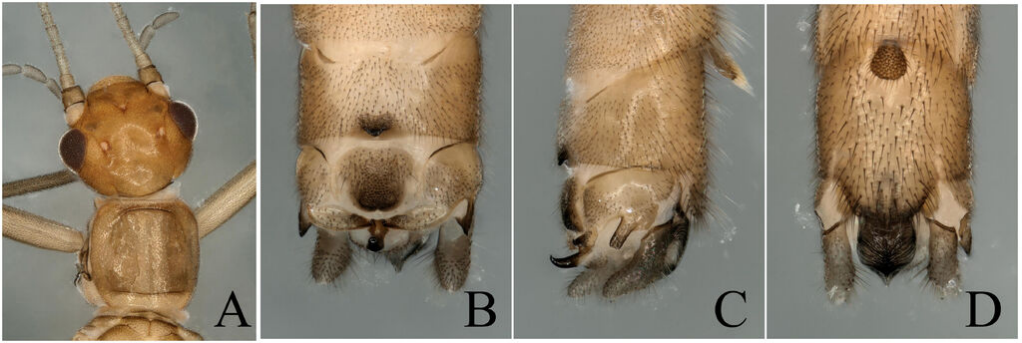
*Rhopalopsolewuyishanensis* Yang & Du, 2021. **A** Male head and pronotum, dorsal view; **B** Male terminalia, dorsal view; **C** Male terminalia, lateral view; **D** Male terminalia, ventral view. Modified from Yang et al. (2021).

Recently described species from Wuyi Mountains of Fujian Province. Head brown, wider than pronotum; ocelli pale brown; antennae and palpi light brown. Pronotum brown (Fig. 10A). Tergum 10 bearing a large central plate covered with broad sensilla basiconica patch in the lower half and somewhat less sclerotized in the upper half. Lateral projections of tergum 10 plate-like, extending backwards with rectangular-shaped process (Fig. 10).

##### Type locality

China, Fujian Province (Wuyi Mountains).

#### Distribution

China (Fujian).

### Rhopalopsole trichotoma

Yang & Du, 2021

057F9572-1633-5415-ACFE-84E349C2EE4E

Rhopalopsole trichotma : Yang et al. (2021): 147–148.

#### Materials

- **Type status:** Other material. **Occurrence:** recordedBy: Yang Yu-Ben and Zhu Bin-Qing (ICYZU); individualCount: 3; sex: male; lifeStage: adult; occurrenceStatus: present; **Taxon:** scientificName: *Rhopalopsoletrichotoma* Yang & Du, 2021; kingdom: Animalia; phylum: Arthropoda; class: Insecta; order: Plecoptera; family: Leuctridae; taxonRank: species; taxonomicStatus: accepted; **Location:** continent: Asia; country: China; countryCode: CN; stateProvince: Fujian; locality: Wuyi Mountains,; minimumElevationInMeters: 303.29; verbatimLatitude: 27°39′33″N; verbatimLongitude: 117°57′50″E; **Identification:** identifiedBy: Yang Yu-Ben & Du Yu-Zhou; **Event:** year: 2020; month: 3; day: 19; verbatimEventDate: 19-03-2020; **Record Level:** language: En; institutionCode: ICYZU; basisOfRecord: PreservedSpecimen.

#### Description

##### Diagnosis and remarks

**Figure 11.**
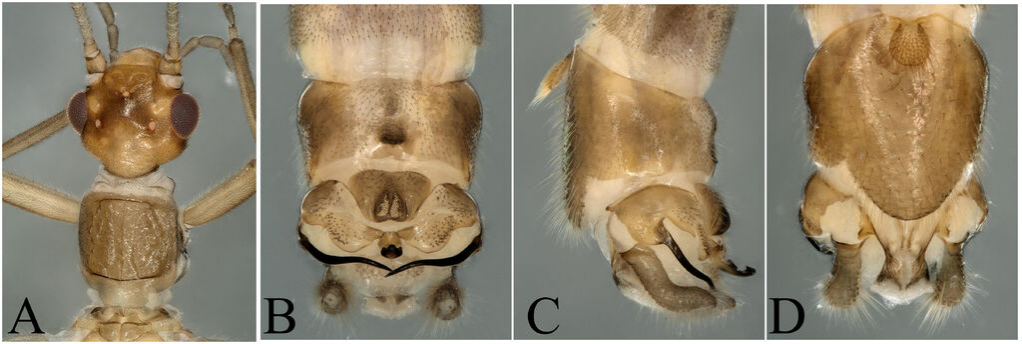
*Rhopalopsoletrichiotoma* Yang & Du, 2021. **A** Male head and pronotum, dorsal view; **B** Male terminalia, dorsal view; **C** Male terminalia, lateral view; **D** Male terminalia, ventral view. Modified from Yang et al. 2021.

Recently described species from Wuyi Mountains of Fujian Province. Head brown, wider than pronotum; ocelli pale brown; antennae and palpi light brown; pronotum brown (Fig. 11A). Tergum 10 bearing a large central plate covered with a sensilla basiconica patch in the two oval areas. Epiproct thick at base, ending in a shallow trilobed process (Fig. 11).

##### Type locality

China, Fujian Province (Wuyi Mountains).

#### Distribution

China (Fujian).

### Rhopalopsole basinigra

Yang & Yang, 1995

109A3C64-C65E-5531-98A9-BC8FD354155D

Rhopalopsole basinigra : Yang and Yang (1995): 61.
Synonym: *Rhopalopsoleduyuzhoui*: Yang and Du (2021b): 487.

#### Materials

- **Type status:** Other material. **Occurrence:** recordedBy: Yang Yu-Ben, Zhu Bin-Qing (ICYZU); individualCount: 3; sex: male; lifeStage: adult; occurrenceStatus: present; **Taxon:** scientificName: *Rhopalopsolebasinigra* Yang & Yang, 1995; kingdom: Animalia; phylum: Arthropoda; class: Insecta; order: Plecoptera; family: Leuctridae; taxonRank: species; taxonomicStatus: accepted; **Location:** continent: Asia; country: China; countryCode: CN; stateProvince: Fujian; locality: Wuyi Mountains (Famuchang); minimumElevationInMeters: 478; verbatimLatitude: 27°36′2″N; verbatimLongitude: 117°45′9″E; **Identification:** identifiedBy: Yang Yu-Ben; **Event:** year: 2021; month: 3; day: 18; verbatimEventDate: 18-03-2021; **Record Level:** language: En; institutionCode: ICYZU; basisOfRecord: PreservedSpecimen.

#### Description

##### Diagnosis and remarks

**Figure 12.**
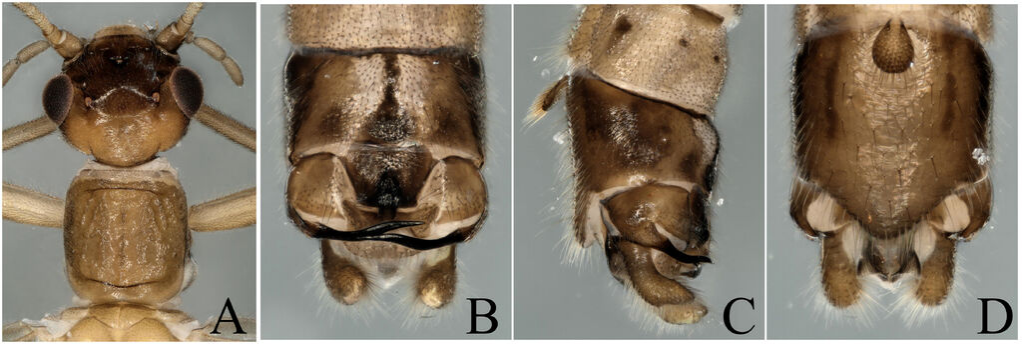
*Rhopalopsolebasinigra* Yang & Yang, 1995. **A** Male head and pronotum, dorsal view **B** Male terminalia, dorsal view; **C** Male terminalia, lateral view; **D** Male terminalia, ventral view.

The report of this species is a new distribution record to Wuyi Mountains and had been redescribed by Qian and Du (2012b). Head dark brown at upper half, brown at lower half, wider than pronotum; ocelli brown; antennae and palpi light brown; pronotum light brown (Fig. 12A). Epiproct thick at base, expanding in top view, ending in a shallow trilobed tip.

##### Type locality

China, Zhejiang (Gutian Mountain).

#### Distribution

China (Zhejiang, Fujian, Guizhou, Shaanxi, Sichuan, Guangxi).

### Rhopalopsole bispina

(Wu, 1949)

1107DF73-DFEA-5F3C-860B-4CEA90E21BBB

Leuctra bispina : Wu (1949): 252.
Rhopalopsole bispina : *Illies (1966)*: 117.

#### Materials

- **Type status:** Other material. **Occurrence:** recordedBy: Yang Yu-Ben, Zhu Bin-Qing (ICYZU); individualCount: 3; sex: male; lifeStage: adult; occurrenceStatus: present; **Taxon:** scientificName: *Rhopalopsolebispina* Wu, 1949; kingdom: Animalia; phylum: Arthropoda; class: Insecta; order: Plecoptera; family: Leuctridae; taxonRank: species; taxonomicStatus: accepted; **Location:** continent: Asia; country: China; countryCode: CN; stateProvince: Fujian; locality: Wuyi Mountains (Famuchang); minimumElevationInMeters: 478; verbatimLatitude: 27°36′2″N; verbatimLongitude: 117°45′9″E,; **Identification:** identifiedBy: Yang Yu-Ben; **Event:** year: 2021; month: 3; day: 18; verbatimEventDate: 18-03-2021; **Record Level:** language: En; institutionCode: ICYZU; basisOfRecord: PreservedSpecimen.

#### Description

##### Diagnosis and remarks

**Figure 13.**
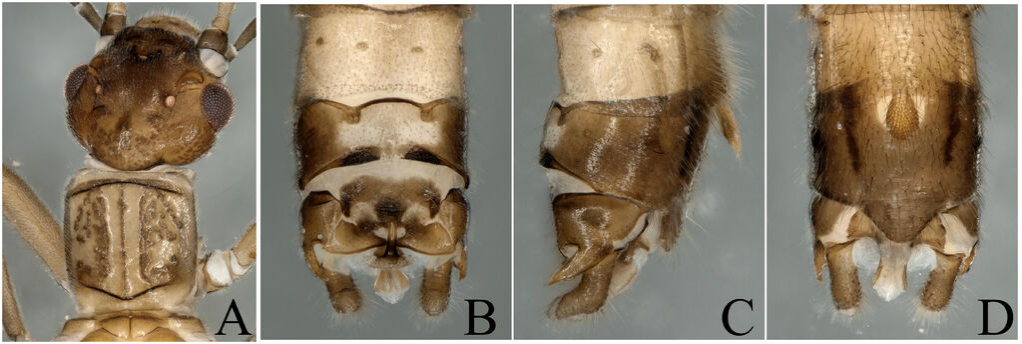
*Rhopalopsolebispina* (Wu, 1949). **A** Male head and pronotum, dorsal view; **B** Male terminalia, dorsal view; **C** Male terminalia, lateral view; **D** Male terminalia, ventral view.

Type of *R.bispina* from 1949 is destroyed or lost. Sivec et al. (2008) place this species in the *R.magnicerca* group, based on a series of specimens from Sichuan Province of southwest China. We also collected this species from the type locality and the morphological characteristics of the topotypes we collected agree with the redescription of Sivec et al. (2008). The hind area of the head has a lot of spots on hind part (Fig. 13). Hind border of tergum 9 with a band of knob-like ornamentation, two round sclerotized spots on the anterior margin. Tergum 10 bearing a central plate, the three elements of which are strongly sclerotized. The middle of anterior margin of transverse plates extend backwards with an irregular ornamentation (Fig. 13).

##### Type locality

China, Fujian Province (Ta–chu–luan, Shao–wu).

#### Distribution

China (Fujian, Sichuan, Guizhou, Zhejiang).

## Identification Keys

### A key to adult males of Leuctridae species from Wuyi Mountains

**Table:** 

1	Cerci deeply forked with a small bulge on dorsal arm (*Paraleuctra*)	*Paraleuctraorientalis* (Chu)
–	Cerci long and not forked (*Rhopalopsole*)	2
2	There are long hairs on the antennae	3
–	There are only short hairs on the antennae	4
3	Lateral projection of tergum 10 ending in a sharp point, overall appearance of subanal lobe is trident-like	*Rhopalopsoleintonsa* Qian & Du
–	Lateral projection of tergum 10 with a bicuspid process, subanal lobe is flat and plate-like	*Rhopalopsolesinensis* Yang & Yang
4	Inner side of lateral projections has a finger-like ornamentation	*Rhopalopsolefengyangshanensis* Yang, Shi & Li
–	Inner side of lateral projections without finger-like ornamentation	5
5	Subanal lobes borered with black spines and short processes on lateral projections	*Rhopalopsolespiniplatta* (Wu)
–	Subanal lobes without black spine	6
6	Tergum 9 with a band of knob-like ornamentations, tergum 10 bearing a central plate, the three elements of which are strongly sclerotized	*Rhopalopsolebispina* (Wu)
–	Tergum 9 evenly sclerotized, with a single ornamentation, tergum 10 does not spilt into three elements	7
7	Lateral projections of tergum 10 plate-like, extending apically with rectangular process	*Rhopalopsolewuyishanensis* Yang & Du
–	Lateral projections of tergum 10 thin, extending backwards with a long and sinuous process	8
8	Epiproct almost as wide as the central plate of tergum 10	*Rhopalopsoleflata* Yang & Yang
–	Epiproct hook-like, ending in a trilobed process	9
9	Tergum 10 is bearing a large central plate covered with sensilla basiconica patch in two oval areas	*Rhopalopsoletrichotoma* Yang & Du
–	Tergum 10 without sensilla basiconica patch	10
10	Epiproct stocky, terminating in a trilobed tip, the middle is round, two other lobes are corners of epiproct	*Rhopalopsoleyangdingi* Sivec & Harper
–	Epiproct thick at base, expanding in top view, ending in a shallow trilobed tip	*Rhopalopsolebasinigra* Yang & Yang

## Discussion

In this study, we found that there are twelve species of Leuctridae recorded in Wuyi Mountains (Table 1). These records include two genera and five new distribution records species: one species of the genus *Paraleuctra*: *P.orientali*s; and eleven species of the genus *Rhopalopsole*, including five new distribution records to Wuyi Mountains: *R.fengyangshanensis*; *R.sinensis*; *R.yangdingi*; *R.flata*; *R.basinigra*. For the composition of Leuctridae in Wuyi Mountains, four endemic species are included: *R.spiniplatta*, *R.recurvispina*, *R.trichotoma*, *R.wuyishanensis* and eight species with different distribution places. These data directly show the diversity of Leuctridae in Wuyi Mountains. We think that this phenomenon mainly comes from two reasons:

Firstly, in terms of history and formation, the Wuyi Mountains were originally a plain of denudation. It developed into a lake basin by the subsidence of the Earth's crust in the early Cretaceous period. The thickness of the deposit in the lake has reached 1500 m. Owing to uplifting in the late Tertiary period, a monoclinic block mountain was formed. The Wuyi Mountains have developed into the following morphological types under the control of geological structures and morphological development: cuesta, block mountain, hill, gentle hill, flat-bottomed alley valley, depressed valley, terrace etc (Liu 1984). We think the long history and complex landform of Wuyi Mountains are important reasons for the diversity of Leuctridae in Wuyi Mountains.

Secondly, in terms of biogeography, Wuyi Mountains are located in the north Palaearctic Region and the edge of the south Oriental Region, in the south of Chongan County, Fujian Province. They cover an area of 60 km^2^ and the altitude is between 180 m and 720 m. They have a unique geographical location, complex landform and superior climatic conditions (Shen 2015). They belong to the middle subtropical monsoon climate and the zonal vegetation is well preserved. The length of the famous Jiuqu River is 9.5 km. The Jiuqu River developed mainly along east-west and north-south strike faults and so, it is a very meandering river, thousands of streams having been formed, based on this river, providing a good ecological environment for Leuctridae species.

Finally, although we have found twelve species in Wuyi Mountains, we still believe that many species have not been reported. In the future, we will continue to investigate the biodiversity of Wuyi Mountains with the expectation of finding more mysterious species.

## Supplementary Material

XML Treatment for Paraleuctra orientalis

XML Treatment for Rhopalopsole recurvispina

XML Treatment for Rhopalopsole spiniplatta

XML Treatment for Rhopalopsole fengyangshanensis

XML Treatment for Rhopalopsole intonsa

XML Treatment for Rhopalopsole sinensis

XML Treatment for Rhopalopsole yangdingi

XML Treatment for Rhopalopsole flata

XML Treatment for Rhopalopsole wuyishanensis

XML Treatment for Rhopalopsole trichotoma

XML Treatment for Rhopalopsole basinigra

XML Treatment for Rhopalopsole bispina

## Figures and Tables

**Table 1.** Leuctridae from Wuyi Mountains.

**Species name**	**Genus**	**Type locality**	**Distribution**	**Figures**	**Notes**
*P.orientalis* (Chu, 1928)	*Paraleuctra*	China, Zhejiang Province (Lin–an)	China (Henan, Anhui, Zhejiang, Fujian, Hubei, Hunan, Shaanxi, Sichuan, Yunan, Gansu); Russia (Siberia)	Fig. 1	The colour and sclerotized patterns bear some variations with the original descriptions, we think the variations are intraspecific
*R.recurvispina* (Wu, 1949), nom. dubium	*Rhopalopsole*	China, Fujian Province (Ta–chu–luan, Shao–wu)	China (Fujian)	Fig. 2	We regard *R.recurvispina* as a nomen dubium after we failed many times to collect this species.
*R.spiniplatta* (Wu, 1949)	*Rhopalopsole*	China, Fujian Province (Ta–chu–luan, Shao–wu)	China (Fujian)	Fig. 3	Type of *R.spiniplatta* from 1949 is destroyed or lost. Collecting additional material from the type locality could resolve the exact identity of this species.
*R.fengyangshanensis* Yang, Shi & Li, 2009	*Rhopalopsole*	China, Zhejiang Province (Fengyang Mountain, Fengyanghu)	China (Zhejiang, Fujian, Jiangxi)	Fig. 4	*R.fengyangshanensis* is a new distribution record to Wuyi Mountains.
*R.intonsa* Qian & Du, 2012	*Rhopalopsole*	China, Jiangxi Province (Wuyi Mountains)	China (Zhejiang, Jiangxi)	Fig. 6	We found the long hairs on the antennae of this species.
*R.sinensis* Yang & Yang, 1993	*Rhopalopsole*	China, Guizhou Province (Maolan)	China (Guangdong, Zhejiang, Fujian, Hubei, Hunan, Henan, Guizhou, Jiangxi, Shaanxi, Sichuan, Guangxi, Ningxia, Yunnan); Vietnam	Fig. 7	*R.sinensis* is a new distribution record to Wuyi Mountains.
*R.yangdingi* Sivec & Harper, 2008	*Rhopalopsole*	China, Jiangxi Province (Dayue Mountain)	China (Fujian, Jiangxi)	Fig. 8	*R.yangdingi* is a new distribution record to Wuyi Mountains.
*R.flata* Yang & Yang, 1995	*Rhopalopsole*	China, Zhejiang Province (Baishanzu Mountain)	China (Guangdong, Zhejiang, Anhui, Fujian)	Fig. 9	*R.flata* is a new distribution record to Wuyi Mountains.
*R.wuyishanensis* Yang & Du, 2021	*Rhopalopsole*	China, Fujian Province (Wuyi Mountains)	China (Fujian)	Fig. 10	Recently described species from Wuyi Mountains.
*R.trichotoma* Yang & Du, 2021	*Rhopalopsole*	China, Fujian Province (Wuyi Mountains)	China (Fujian)	Fig. 11	Recently described species from Wuyi Mountains.
*R.basinigra* Yang & Yang, 1995	*Rhopalopsole*	China, Fujian Province (Ta–chu–luan, Shao–wu)	China (Zhejiang, Fujian, Guizhou, Shaanxi, Sichuan)	Fig. 12	*R.basinigra* is a new distribution record to Wuyi Mountains.
*R.bispina* (Wu, 1949)	*Rhopalopsole*	China, Zhejiang Province (Gutianshan Mountain)	China (Zhejiang, Fujian)	Fig. 13	The morphological characteristics of the topotypes we collected also agree with the redescription of Sivec et al. (2008).
